# Does Spatial Locative Comprehension Predict Landmark-Based Navigation?

**DOI:** 10.1371/journal.pone.0115432

**Published:** 2015-01-28

**Authors:** Laura Piccardi, Liana Palermo, Alessia Bocchi, Cecilia Guariglia, Simonetta D’Amico

**Affiliations:** 1 Life, Health and Environmental Science Department, University of L’Aquila, L’Aquila, Italy; 2 Neuropsychology Unit, IRCCS, Fondazione Santa Lucia, Rome, Italy; 3 School of Life & Health Sciences, Aston University, Birmingham, United Kingdom; 4 Psychology Department, Sapienza University of Rome, Rome, Italy; University of L'Aquila, ITALY

## Abstract

In the present study we investigated the role of spatial locative comprehension in learning and retrieving pathways when landmarks were available and when they were absent in a sample of typically developing 6- to 11-year-old children. Our results show that the more proficient children are in understanding spatial locatives the more they are able to learn pathways, retrieve them after a delay and represent them on a map when landmarks are present in the environment. These findings suggest that spatial language is crucial when individuals rely on sequences of landmarks to drive their navigation towards a given goal but that it is not involved when navigational representations based on the geometrical shape of the environment or the coding of body movements are sufficient for memorizing and recalling short pathways.

## Introduction

Human beings develop navigational skills gradually and at distinct points in time [1; 2]. During the first year of life, children start developing an awareness of their own motion in space. By the age of 6–9 months, they are able to find their bearings in the environment using only egocentric strategies (turning to the left/right to reach a target: [3–6]). At 11 months they start to use information pertaining to landmarks and landmark arrays [3; 4]. Egocentric information is gradually supplemented with allocentric coding. The relation-place strategies required for cognitive mapping start to develop around 7 or 8 years of age and are fully functioning by the age of 10 [7; 2; 8–10].

Humans develop some specific navigational skills, which are absent in other animal species, and are able to use verbal language to represent the environment. The relationship between language and spatial representation (particularly in the use of landmarks) has already been underlined [11; 12], but its role in human navigation is still not clear. Effects of language on navigation might represent a subset of a broader class of experiences that influence navigation through domain-general mechanisms, or these effects might be specific to the linguistic domain. In either case, the mechanisms through which language plays a role in spatial cognition have never been specified. Language might direct attention to, enhance memory for or integrate distinct sources of information about the environment (e.g., [13–17]).

According to the specificity hypothesis [18; 19] a relationship exists between the emergence of linguistic and nonlinguistic skills that rely on shared basic knowledge. Balcomb et al. [20] used an adapted Morris water maze and the MacArthur Communicative Development Inventory to investigate the relationship between early spatial language and navigational skills in 16- to 24-month-old children. Their results showed that older children demonstrated more spatial searching and better place-learning skills as well as greater overall expressive vocabulary. It has also been shown that the acquisition of locative prepositions (i.e. in/out) is correlated with place learning skills. Although none of the children said “next to” or “by” to describe relationships between objects in a horizontal plane that were useful for reaching the goal in the navigational tasks, the authors suggested that a child who notices a spatial construct is also more likely to notice its label and vice-versa.

Linguistic encoding is instrumental in supporting children’s ability to represent left–right relations in visual memory tasks [21; 14]. Also, data collected in clinical populations, such as children with William Syndrome, a rare genetic disease characterized by impaired spatial cognition, seem to suggest that linguistic spatial concepts have an important role in navigational development. For example, children with Williams syndrome are deficient in using prepositions (e.g., [22; 23]). Furthermore, Pyers et al. [12] reported that adults who are impaired in the use of left–right spatial language perform reorientation tasks deficiently.

In the present study, we investigated the role of spatial locative comprehension in a sample of typically developing children between 6 and 11 years of age. At variance with Balcomb et al. [20], we directly tested the children’s ability to understand spatial concepts and compared their grammar skills with their ability to learn pathways. We hypothesized that language plays a specific role in navigation when environments contain elements/landmarks that can be verbally labelled. To test this hypothesis we used two conditions in the pathway learning task, that is, with and without landmarks. Furthermore, as gender differences have been demonstrated in the ability of young adults to learn sequences in the WalCT [24], we aimed to verify whether these differences are already present in children.

## Methods

### Participants

A sample of 81 typically developing children (48 M and 33 F) took part in the study. Ages ranged from 6 years and 1 month to 11 years and 2 months (6yrs: 6M, 4F; 7yrs: 11M, 5F; 8yrs: 9M, 7F; 9yrs: 10M, 6F; 10yrs: 8M, 6F; 11yrs = 4M, 5F). Mean age was 103.17 months (SD = 16.90 months) in male children and 109.18 months (SD = 16.54 months) in female children. All were pupils at the ‘Istituto Bambin Gesù’ primary school in Rieti (Italy) and were Italian native speakers. Foreign children and those with learning difficulties and other neurodevelopmental diseases (as reported by their teachers or families) were not included in the study. None of the children included in the study had primary visual or hearing impairments, had been diagnosed with a neurological condition or had ever shown any emotional or behavioural problem. To determine the general cognitive level, all children were administered Raven’s Coloured Progressive Matrices [25; 26]; no difficulty in clear-thinking ability emerged.

The study was approved by the local ethics committee of Psychology Department of “Sapienza” University of Rome in accordance with the Declaration of Helsinki. A signed consent form was obtained from parents and an assent from each child. Children were tested individually in a quiet room of the primary school. Further, we obtained informed consent for publication of the child's photograph from his parents.

### Instruments and procedure

**Figure 1:**
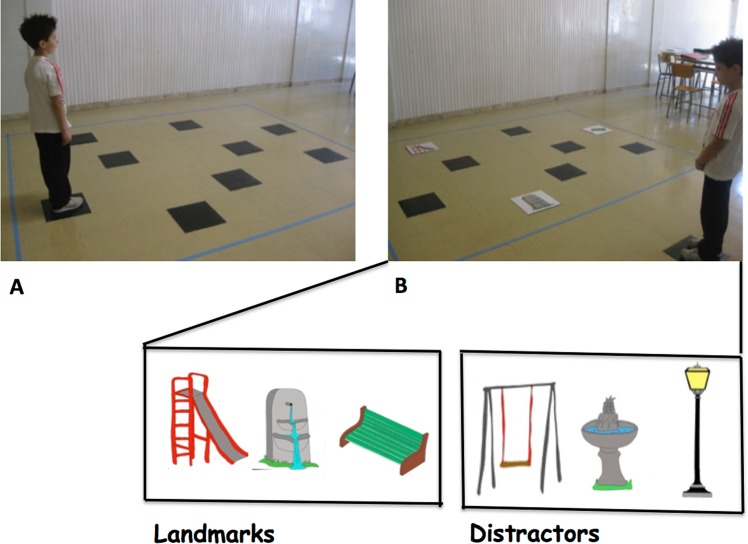
WL-WalCT (A) and L-WalCT (B). The bottom of the figure shows landmarks present in the landmark condition and distractors shown during the landmark recognition task.

**Walking Corsi Test (WalCT: [24; 27; 28]).** The Walking Corsi Test (WalCT) is a larger version (3 × 2.5 m; scale 1:10) of the Corsi Block Tapping Test (CBT) [39]). It has primarily been used for experimental and clinical purposes [24; 28; 29; 30; 31; 32; 33; 34; 35; 36; 37; 38] to investigate topographical memory by asking individuals to reproduce a previously observed pathway. The WalCT is set up in an empty room. It is composed of nine black squares (30 × 30 cm) that are placed on the floor. This test is scaled to the standard CBT. The starting point is the black square located outside the layout (see Fig. 1A).

In the WalCT, the examiner walks and stops on a series of squares. The subject has to actually walk, reach different locations and reproduce the sequence demonstrated by the examiner. In this study, two aspects of topographical long-term memory were assessed: learning (TL) and delayed recall (TDR) in two different conditions, namely, with landmarks (L-WalCT) and without landmarks (WL-WalCT). The only difference between the L-WalCT and the WL-WalCT condition was the presence in the L-WalCT of the pictures of three landmarks (a fountain, a bench and a slide: see Fig. 1B) placed on three black squares.

In the TL, the children had to learn a fixed supra-span sequence, which was calculated according to their chronological age by considering the span + 2 according to the median span sample of Piccardi et al. [28]’s study. Specifically, 6-year-old children had to learn a 4-block sequence (because at this age the median span was 2) and children above 6 years of age had to learn a 5-block sequence, (because at this age the median span was 3). In each trial, after the examiner presented the sequence the child was invited to step on the carpet to reproduce it stepping out of the carpet when he/she had finished. In each trial number of correct black squares reproduced in the sequence is calculated for the final score, but no feedback about the correctness of performance was provided. Learning criterion (indicating that learning was achieved) corresponds to three consecutive correct reproductions of the sequence; in case the child did not reach the learning criterion the sequence was repeated for a maximum of 18 trials. The learning score was calculated by attributing one point for each square correctly walked until the criterion was reached; the score corresponding to correct performance (e.g., 4 for 6yrs and 5 for older children) of the remaining trials was added to this score (up to the 18th; maximum score: 72 for 6-year-old children and 90 for older children). Five minutes later, the TDR was administered. The examiner asked the participant to reproduce the previously learned four/five-block sequence in a one-shot trial. The score was the number of squares correctly reproduced.

**Figure 2:**
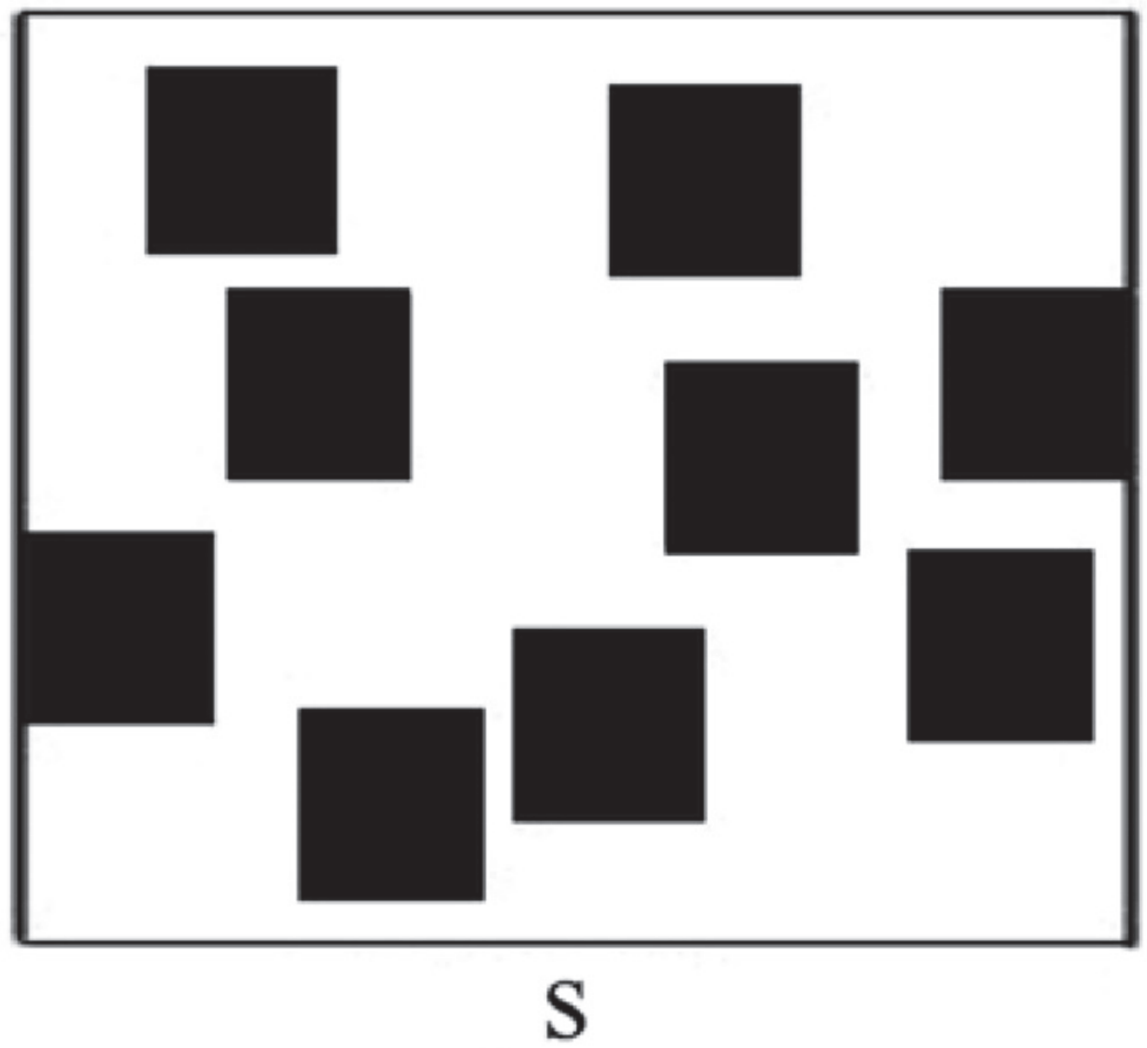
Outline map of the WalCT on which the children had to draw the pathway and in the L-WalCT to place the landmarks.

At the end of the L-WalCT the children were shown 6 items (3 landmarks and 3 distractors: Maximum score: 6; See Fig. 1B). They had to indicate which of the landmarks were present in the L-WalCT. Then they were asked to place them on an outliner of the WalCT (Maximum Score: 3; see Fig. 2). Finally, at the end of both the L-WalCT and the WalCT the children had to use a felt tip marker to retrace the pathways they had learned in the two conditions on the outline of the WalCT (Maximum Score: 4 or 5 according to chronological age of children).

**Test for Reception of Grammar (TROG: [40]; Italian version: [41]).** We tested verbal comprehension of spatial locatives using the TROG, which assesses grammatical comprehension from age 4 to adulthood. Each test stimulus is presented in a four-picture multiple-choice format with lexical and grammatical foils. Grammatical complexity increases consistently, from locative structure to active, passive, negative, dative and relative clauses. For experimental purposes we selected only the spatial locative sentences (14 sentences); they included locative topological elements (below/above, up/down, in/out and near/far) and prospective locative elements (in front of/behind, from/to and between). The participants’ task was to select the picture that matched a sentence spoken by the examiner; in the case of errors the same sentence was re-spoken. The score was 1 if the answer was immediately correct, 0.5 if the answer was correct after the second presentation and 0 if the answer was wrong.

The administration order of the WL-WalCT, L-WalCT and TROG was counterbalanced across the children in each group.

### Statistical analysis

Since different-aged children learned pathways of different lengths, to compare performances at different ages we transformed the L/WL-WalCT scores into percentages.

Pearson correlations were performed to explore the relationship between the ability to comprehend spatial locatives (TROG), to learn a pathway with and without landmarks (TL L-WalCT and TL WL-WalCT), to retrieve it after a delay (TDR L-WalCT and TDR WL-WalCT), to recognize and locate landmarks on the outline and to track the learned pathway on the outline.

Separate simple linear regression analyses were performed to investigate the degree to which participants’ chronological age and spatial locative skills comprehension (TROG) contributed to predicting their performance in learning (TL L-WalCT and TL WL-WalCT) and delayed recall of pathways (TDR L-WalCT and TDR WL-WalCT), as well as in recognizing and locating landmarks and tracking pathways on the outline.

Single-way ANOVAs were performed to detect differences in age and TROG performance between male and female participants. Single-way ANOVAs were also performed to verify the presence of gender differences in the ability to recognize landmarks and to locate them on the outline of the L-WalCT. Finally, to verify whether gender differences had a role pathway learning in the L-WalCT and the WL-WalCT, we performed a 2 × 2 mixed ANOVA (Gender × TL-WalCT conditions) in which gender was considered as the independent variable and performance on the TL/L-WalCT and TL/WL-WalCT as the dependent variable. We also performed a 2 × 2 mixed ANOVA to determine whether there were gender differences in the delayed recall of a pathway in the L-WalCT and the WL-WalCT and in the drawing of a pathway in the L-WalCT and the WL-WalCT outline.

The alpha level was set at .05.

## Results

### Demographic variables and administration order

The samples of male and female children did not differ for age (F_(1, 79)_ = 2.52, p = .11, partial eta-squared = .03) and performance on the TROG (F_(1, 79)_ = 2.09, p = .15; partial eta-squared = .02). See Table 1 for means and SD.

**Table 1:** Means and SDs of the experimental tasks.

Task	Females	Males
TL WL-WalCT	94.54 (8.80)	91.89 (14.63)
TL L-WalCT	97.27 (4.61)	95.87 (11.94)
TDR WL-WalCT	98.94 (4.64)	95.83 (20.19)
TDR L-WalCT	98.78 (6.96)	94.69 (20.97)
Drawing WL-WalCT	73.64 (33.31)	84.74 (29.81)
Drawing L-WalCT	73.94 (36.67)	71.46 (36.20)
Landmark recognition	97.47 (6.07)	93.40 (14.88)
Landmark position	80.81 (26.39)	70.83 (31.97)
TROG	.20 (.50)	.44 (.86)

In order to rule out an effect related to the administration order of L-WalCT and the WL-WalCT, we counterbalanced presentation order across the participants in each group. Then we performed a 2 (type of test: L-WalCT vs WL-WalCT) × 2 (administration order: L-WalCT- WL-WalCT vs WalCT- L-WalCT) mixed ANOVA on learning, delayed recall and drawing of pathway scores.

For the learning condition, the analysis revealed a main effect of the test (F(1,79) = 11.36, p < .001, partial eta-squared = .13) but not a significant effect of the administration order (F(1,79) = .47, p = .49, partial eta-squared = .01), or a significant interaction between type of test and administration order (F(1,79) = 0.41, p = .52, partial eta-squared = .01).

For the delayed recall condition, the analysis did not show a main effect of the test (F(1,79) = 1.48, p = .70, partial eta-squared = .002), of the administration order (F(1,79) = 1.61, p = .21, partial eta-squared = .02), or a significant interaction between type of test and administration order (F(1,79) = 0.10, p = .75, partial eta-squared = .001). Similarly, for the drawing of pathway, the analysis did not show a main effect of the test (F(1,79) = 3.09, p = .08, partial eta-squared = .04), of the administration order (F(1,79) = .90, p = .21, partial eta-squared = .01), or a significant interaction between type of test and administration order (F(1,79) = 3.15, p = .08, partial eta-squared = .04).

### Correlations among variable

We found that chronological age was correlated with both the ability to learn a pathway in the Landmark condition (r = .274, p<.01; see Table 2) and the ability to comprehend spatial locatives (r = .308, p = .005; see Table 2). The ability to learn in the WL-WalCT and the L-WalCT was also correlated with the ability to draw the previously learned pathway on the outline (r = .364, p = .001 and r = .298, p<.01, respectively; see Table 2) as well as with recalling it after a delay (r = .815, p<.001 and r = .597, p<.001, respectively; see Table 2). Further, learning and delayed recall in the two conditions were correlated with each other (r = .683, p<.001 and r = .430, p<.001, respectively; see Table 2), that is, faster learning corresponded with better recall. In addition, the ability to locate landmarks on the outline of the WalCT was correlated with both the ability to draw the path on the outline (r = .299, p<.01) and the ability to learn the path (r = .221, p<.05) in the L-WalCT (see Table 2 for further details on correlations among tests).

**Table 2:** Pearson’s Correlations among tasks and age.

	TL WL-WalCT	TL L-WalCT	Drawing WL-WalCT	Drawing L-WalCT	TDR WL-WalCT	TDR L-WalCT	Landmark recognition	Landmark position	TROG	Age
TL WL-WalCT	1	.683**	.364**	.192	.815**	.220*	.074	.069	-.140	.203
TL L-WalCT	.683**	1	.387**	.298**	.868**	.597**	.102	.221*	-.229*	.274*
Drawing WL-WalCT	.364**	.387**	1	.287**	.441**	.136	-.043	.074	-.070	-.061
Drawing L-WalCT	.192	.298**	.287**	1	.218	.187	.077	.299**	-.028	.077
TDR WL-WalCT	.815**	.868**	.441**	.218	1	.430**	.060	.160	-.181	.189
TDR L-WalCT	.220*	.597**	.136	.187	.430**	1	.053	.159	-.522**	.166
Landmark recognition	.074	.102	-.043	.077	.060	.053	1	.131	.073	.214
Landmark position	.069	.221*	.074	.299**	.160	.159	.131	1	-.015	.157
TROG	-.140	-.229*	-.070	-.028	-.181	-.522**	.073	-.015	1	-.308**
Age	.203	.274*	-.061	.077	.189	.166	.214	.157	-.308**	1

** p < .01

* p < .05

### Effect of Age and spatial locative comprehension on performance

**Figure 3:**
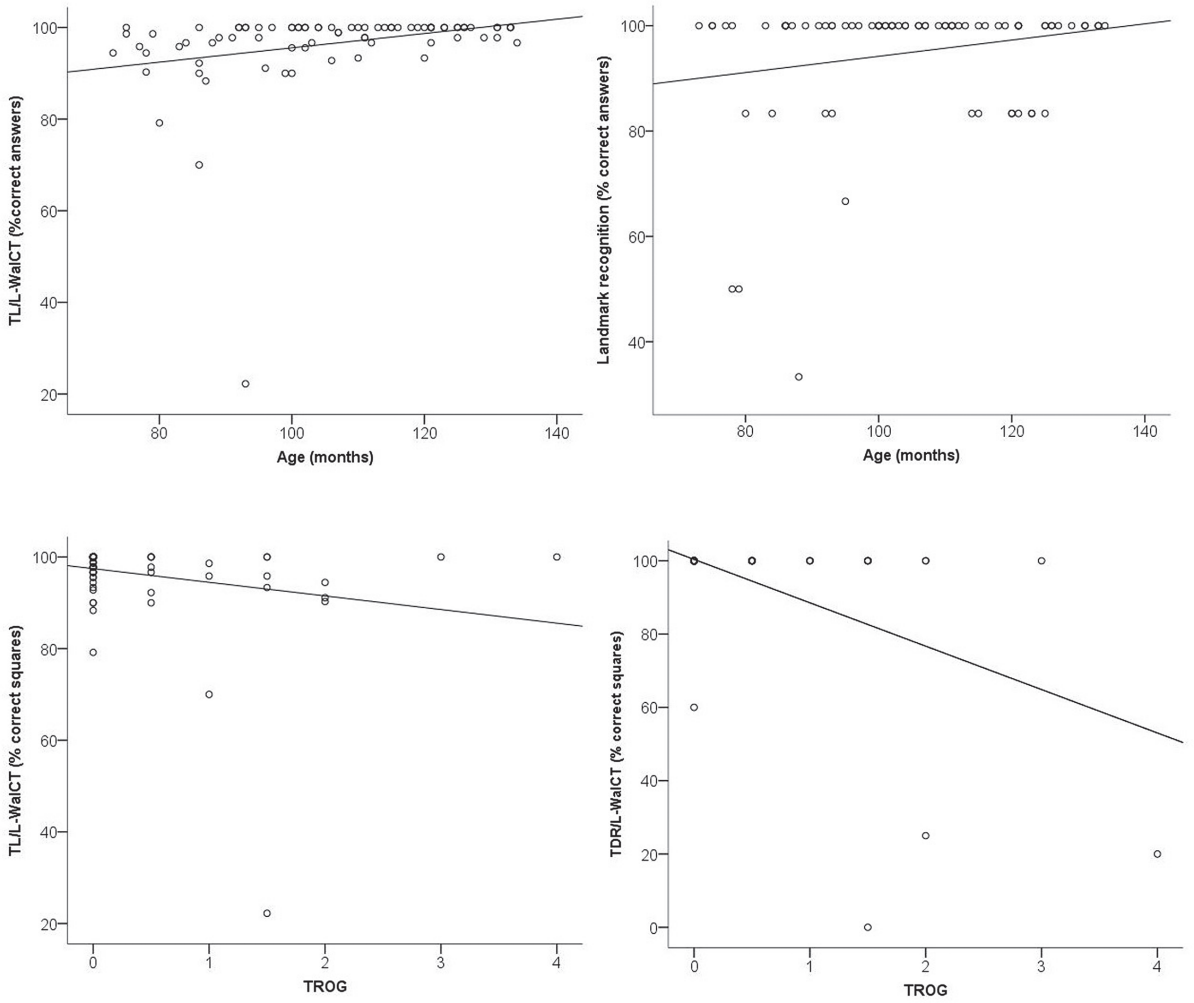
Regression plots for TL/LWalCT (% CR) vs. Age (months) (upper-left); Landmark recognition (% CR) vs. Age (months) (upper-right); TL/LWalCT (% CR) vs. TROG (% errors) (bottom-left) and TDR/LWalCT (% CR) vs. TROG (% errors) (bottom-right).

We also performed simple separate linear regression analyses to investigate the degree to which the chronological age of participants and their ability to understand spatial locatives contributed to predicting their performance in the learning and delayed recall of pathways as well as in recognizing and locating landmarks and in drawing pathways on the outline. The regression analyses showed that the participants’ chronological ages predicted only their ability to learn a pathway in the L-WalCT and to recognize the landmarks (TL/L-WalCT: R = 0.274, R^2^ = 0.075; F_(1, 80)_ = 6.426, p<.01; Landmarks recognition %: R = 0.214, R^2^ = 0.046; F_(1, 80)_ = 3,783, p = .05). TROG performance predicted only the ability to learn and to recall a pathway in the L-WalCT (TL/L-WalCT: R = 0.229, R^2^ = 0.052; F_(1, 80)_ = 4.364, p = .01; TDR/L-WalCT: R = 0.522, R^2^ = 0.273; F_(1, 80)_ = 29.650, p < .001). See Fig. 3.

### Gender differences

In order to verify whether gender differences were already present in children and affected their learning of a pathway in the L-WalCT and the WL-WalCT, we performed a 2 × 2 mixed ANOVA (Gender × TL-WalCT conditions) in which gender was considered as the independent variable and performance on the TL/L-WalCT and theTL/WL-WalCT as the dependent variable. The ANOVA showed a non significant main effect of gender (F_(1, 79)_ = .77, p = .38, partial eta-squared = .01), a significant main effect of conditions (i.e., the TL/L-WalCT was more difficult than the TL/WL-WalCT; F_(1, 79)_ = 10.19 p<.01, partial eta-squared = .11; TL/L-WalCT: M = 92.97, SD = 12.58; TL/WL-WalCT: M = 96.44, SD = 9.63) and a non significant Gender × Conditions interaction (F_(1,79)_ = .35, p = .55, partial eta-squared = .004), suggesting the absence of any gender difference in 6–10 year-old children. Results of the 2 × 2 mixed ANOVA, which was carried out to investigate the presence of gender differences in the delayed recall of a pathway on L-WalCT and the WL-WalCT, showed no significant main effects of the gender (F_(1, 79)_ = 1.34, p = .25, partial eta-squared = .02), conditions (F_(1, 79)_ = .11 p = .74, partial eta-squared = .001) or Gender × Conditions interaction (F_(1, 79)_ = .06, p = .80, partial eta-squared = .00).

Furthermore, another 2 × 2 mixed ANOVA (Gender × Drawing of a Pathway in the L and WL outline) showed no main effects of gender (F_(1, 79)_ = .49, p = .49, partial eta-squared = .01), condition (F_(1, 79)_ = 2.02, p = .16, partial eta-squared = .02) or interaction (F_(1, 79)_ = 2.22, p = .14, partial eta-squared = .03).

Finally, the single-way ANOVAs, which were performed to detect gender differences in the ability to recognize landmarks and to locate them on the outline of the L-WalCT, showed no significant gender differences in Landmark location on the outline (F_(1, 79)_ = 2.18, p = .14, partial eta-squared = .03) or Landmark recognition (F_(1, 79)_ = 2.21, p = .14, partial eta-squared = .03).

See Table 1 for means and SD of the experimental tasks.

## Discussion

Starting from the hypothesis that spatial linguistic competence and non-linguistic skills rely on basic shared knowledge, we investigated the role of spatial locative comprehension in learning and delayed recall of a pathway when landmarks were present or absent.

We hypothesized that acquired competencies in spatial locative understanding would positively affect the ability to learn and retrieve navigational information when visual stimuli that can be used as reference points for navigation (i.e., landmarks) are present in the environment and mostly when landmark-based navigation is required.

Developmental studies have shown that children acquire the ability to re-orient by relying on the geometric shape of the environment very early (i.e., at about 18 months) and well before they formally learn to correctly use the terms left and right [42]. Although young children are able to learn and recognize landmarks in a relatively small environment, they acquire the ability to use landmarks for re-orienting only in later developmental stages, when their language abilities also improve ([43]; for a review see [44]). Studies on the acquisition of spatial terms report that the age of acquisition is around 5–6 years of age but that high individual variability is observed; in some cases acquisition takes place over a protracted period of time [45]. Analogously, the ability to process complex environmental representations, which bind together the processing of geometric features, landmark identities and directional information, seems to start developing around 6–8 years of age depending on both the development of visuospatial processing abilities and language.

According to Dessalegn and Landau [21] and Haun and co-workers [14], linguistic encoding is instrumental in supporting children’s ability to represent left–right relations in visual memory tasks. We believe that it is a basic pre-requisite that allows children to develop cognitive maps that are not limited to the representation of geometric features of the environment, but represent visual cues and landmarks as well as their relative positions. The present results show that an evolved ability to understand spatial grammar is related to children’s better ability to locate landmarks on a map. This demonstrates that children with higher levels of spatial grammar understanding are also able to develop more complex cognitive maps.

The role of locative terms and spatial grammar in navigation was confirmed by some clinical data showing that in Williams Syndrome the presence of deficits in using prepositions affects navigational skills (e.g., [22; 23]).

However, contrasting results were obtained in studies on adults with acquired language disorders. Studies in healthy participants (college students) performed by Hermer-Vazques et al. [46] demonstrated a strong effect of verbal interference in a re-orientation task in which verbal interference suppressed the ability to identify an object with respect to the landmark, which did not affect their ability to use environmental geometric information. Instead, studies in brain-damaged patients showed unimpaired re-orientation performances in adults with language deficits following left brain damage (i.e., aphasia) even when landmarks were present (e.g. [47–49]). Bek et al. [50] studied patients with severe aphasia and observed that neither lexical nor syntactic deficits compromised their spatial representations. It should be underlined, however, that due to the complexity of navigational tasks studies performed in left brain-damaged patients explicitly excluded patients affected by comprehension deficits [47–49]; thus, they excluded patients affected by specific problems in understanding and using spatial locatives. However, the possible role of spatial language in navigation is supported by the Barkas et al. [51]’s observation that found the level of impairment of spatial tasks in patients with selective temporal resections is dependent on how easily cues are verbalized. It is also possible that spatial language has a different role at different ages of development, as its role is important only during the development of navigational skills and as language is not involved in representing navigational information after navigational skills have fully been acquired.

Even if the sample wideness requires some cautious in drawing conclusions, present data clearly suggest that the development of spatial language is related to navigational memory only when landmarks are present in the environment. Balcomb et al. [20] found that in children proficiency in finding a target increased concomitantly with expressive vocabulary. These authors also found that the acquisition of spatial prepositions predicts children’s ability to learn a spatial location in the environment.

Our data support the findings of Balcomb et al. [20] and suggest that the role of language is strictly related to the navigational task to be performed. Indeed, it has been found that the level of spatial language does not affect the ability to learn a pathway, reproduce it after a delay or draw it when landmarks are absent and the only features represented refer to the shape of the environment, geometric and metric features about the location of the squares and the direction and length of individual movements in the environment. It is noteworthy, however, that most of everyday navigation is performed in environments that are full of visual cues and landmarks, in which the role of spatial language cannot be underestimated.

Another result of the present study was the absence of gender differences in this sample. Our previous studies in adults showed the presence of gender differences: men performed better in the WalCT than women and used the same strategy to solve a navigational memory and a visuo-spatial task (CBT: [39]). By contrast, women were significantly better on the WalCT than the CBT [24]. In a recent study, Piccardi et al. [33] also observed that women’s performance was significantly different from that of men when they had to consider only spatial location or sequential order. This suggests that in a walking space women remember sequential order and men remember mainly spatial location. These data suggest that women prefer a mental representation of the environment based on a sequential record of steps, which lead from a starting point through landmarks in which each step is coupled with self-instruction resulting from personal experience, which leads to the next step [52]. Gender-related effects show a male advantage in navigation [53] and a female advantage when navigational tasks require language skills [54]. However, during development gender differences emerge rarely and results are largely conflicting. Farrell Pagulayan et al. [55] found no gender differences in the spatial span capacity of 340 participants aged 6 to 13 years, and Nichelli et al. [56] observed no differences in performance between boys and girls aged 5–14 years. León et al [57] found gender differences in 6–8-year-old groups only in a spatial task requiring reference-memory; they observed no difference in the working-memory version of the same task. Their results lend support to the idea that gender differences in the navigational domain may be related to task requirements (i.e., [58]) above and beyond differences related to time of brain structure maturation in boys and girls (see for instance [57]). In contrast to these results and in line with a contribute in detecting differences due to the task requirements, Piccardi et al. [28] observed that girls outperformed boys in navigational working memory.

The absence of gender differences could be due to the sample distribution (i.e., different amounts of M and F) or to the age groups included in the present study, which have a large range, but is limited to prepubescent period. Furthermore, it is noteworthy the age range included in the study navigational memory skills are still very limited masking the presence of gender differences. Currently, there is a debate over the role of hormones in determining sex differences, because the latter appear in early adolescence and remain throughout adulthood (see for instance, [59; 60]).

In conclusion, language, in particular the comprehension of spatial concepts, seems crucial when human beings navigate by using landmarks. In any case, human navigation is a very complex ability that relies on very different cognitive competences to assure success in wayfinding and in spatial re-orientation.
